# Extracellular matrix stiffness controls cardiac valve myofibroblast activation through epigenetic remodeling

**DOI:** 10.1002/btm2.10394

**Published:** 2022-08-22

**Authors:** Cierra J. Walker, Dilara Batan, Carrie T. Bishop, Daniel Ramirez, Brian A. Aguado, Megan E. Schroeder, Claudia Crocini, Jessica Schwisow, Karen Moulton, Laura Macdougall, Robert M. Weiss, Mary A. Allen, Robin Dowell, Leslie A. Leinwand, Kristi S. Anseth

**Affiliations:** ^1^ Materials Science and Engineering Program University of Colorado Boulder Boulder Colorado USA; ^2^ BioFrontiers Institute, University of Colorado Boulder Boulder Colorado USA; ^3^ Biochemistry Department University of Colorado Boulder Boulder Colorado USA; ^4^ Chemical and Biological Engineering Department University of Colorado Boulder Boulder Colorado USA; ^5^ Molecular, Cellular, and Developmental Biology Department University of Colorado Boulder Boulder Colorado USA; ^6^ Division of Cardiology University of Colorado Anschutz Medical Campus Aurora Colorado USA; ^7^ Department of Internal Medicine University of Iowa Iowa City Iowa USA

**Keywords:** ATAC‐Seq, epigenetics, hydrogels, myofibroblasts, RNA‐Seq, valve interstitial cells

## Abstract

Aortic valve stenosis (AVS) is a progressive fibrotic disease that is caused by thickening and stiffening of valve leaflets. At the cellular level, quiescent valve interstitial cells (qVICs) activate to myofibroblasts (aVICs) that persist within the valve tissue. Given the persistence of myofibroblasts in AVS, epigenetic mechanisms have been implicated. Here, we studied changes that occur in VICs during myofibroblast activation by using a hydrogel matrix to recapitulate different stiffnesses in the valve leaflet during fibrosis. We first compared the chromatin landscape of qVICs cultured on soft hydrogels and aVICs cultured on stiff hydrogels, representing the native and diseased phenotypes respectively. Using assay for transposase‐accessible chromatin sequencing (ATAC‐Seq), we found that open chromatin regions in aVICs were enriched for transcription factor binding motifs associated with mechanosensing pathways compared to qVICs. Next, we used RNA‐Seq to show that the open chromatin regions in aVICs correlated with pro‐fibrotic gene expression, as aVICs expressed higher levels of contractile fiber genes, including myofibroblast markers such as alpha smooth muscle actin (αSMA), compared to qVICs. In contrast, chromatin remodeling genes were downregulated in aVICs compared to qVICs, indicating qVICs may be protected from myofibroblast activation through epigenetic mechanisms. Small molecule inhibition of one of these remodelers, CREB Binding Protein (CREBBP), prevented qVICs from activating to aVICs. Notably, CREBBP is more abundant in valves from healthy patients compared to fibrotic valves. Our findings reveal the role of mechanical regulation in chromatin remodeling during VIC activation and quiescence and highlight one potential therapeutic target for treating AVS.

## 1 INTRODUCTION

Aortic valve stenosis (AVS) is a progressive disease affecting approximately 0.4% of the US population.^1^ During AVS, the valve leaflets stiffen, alter blood flow through the heart, and can eventually lead to heart failure.^2^ In the past, AVS was viewed as a passive degeneration of the valve, necessitating replacement. Surgical valve repair or valve replacement is the only approved treatments^1^, ^3^, ^4^; however, recent research has revealed the active role of the resident valve cells during AVS. Specifically, valve fibroblasts, also known as valve interstitial cells (VICs), become activated as their microenvironment is altered, and tissue homeostasis is disrupted.^5^ The persistence of activated VICs in diseased valve tissue suggest that epigenetic changes may be occuring during progression of AVS, a concept that presents potential therapeutic opportunities. For example, stenotic valves are associated with an increase in global DNA methylation compared to healthy valves.^6^ Histone modifying proteins, including SIRT1, SIRT6, HDAC1, and HDAC6, are also dysregulated during AVS progression.^7^, ^8^, ^9^, ^10^ Notably, acetylation levels of both histones 3 and 4 are elevated in calcific human aortic valves.^11^ However, the complexity of the in vivo environment during AVS progression has limited the ability to dissect specific mechanisms responsible for these epigenetic changes, impeding progress toward identifying new therapeutic targets.

Biomaterial matrices provide in vitro microenvironments to study VICs in their quiescent and activated states and facilitate novel approaches to investigate epigenetic mechanisms related to molecular changes that occur in VICs during AVS. VICs are responsible for maintaining valve homeostasis and function.^12^ In healthy individuals, VICs are predominately in a quiescent state (qVICs), but in response to an injury, qVICs transition to an activated myofibroblast phenotype (aVICs) to remodel extracellular matrix (ECM).^13^ aVICs promote wound healing by increasing contractile forces, elevating their secretory properties and production of inflammatory molecules, and remodeling ECM.^13^, ^14^ Upon resolution of the injury, aVICs typically de‐activate and return to the qVIC phenotype^15^; however, as AVS progresses, aVICs often persist in the tissue^16^ or even transform into an osteoblast‐like VICs (obVICs).^17^ Recent studies suggest that epigenetic mechanisms are involved in these VIC state transitions. When VICs are cultured on tissue culture plastic in the presence of osteogenic media, histone acetylation promotes the obVIC phenotype.^11^, ^18^ Since increased histone acetylation results in increased chromatin accessibility, these data lead to the hypothesis that differential accessibility of genes, and thereby their transcription, play a role in overall VIC phenotype transitions.

Beyond biochemical cues derived from cells and tissues or delivered in culture media, microenvironmental mechanical cues, such as stiffness, can also initiate the transition of qVICs to aVICs.^15^ Recently, we showed that stiffness can influence VIC epigenetic mechanisms and chromatin landscapes.^19^ Using hydrogels that mimic the matrix stiffness of healthy or diseased valves, respectively, we found that qVICs on soft hydrogels had increased histone deacetylase (HDAC) activity compared to aVICs on stiff hydrogels.^19^ Additionally, aVICs had significantly less chromatin condensation compared to qVICs, suggesting that matrix stiffness likely impacts the qVIC to aVIC transformation via chromatin remodeling. However, it remains unclear how stiffness‐dependent chromatin remodeling and molecular level nuclear changes define the qVIC to aVIC transition.

Here, we aimed to dissect the role of epigenetics and chromatin remodeling during mechanically induced VIC activation and its effects on gene expression. We cultured VICs on soft or stiff hydrogels to obtain populations of qVICs and aVICs, respectively. We found that qVICs and aVICs showed different epigenetic modifications as measured by immunostaining and global chromatin accessibility measured by ATAC sequencing (ATAC‐Seq). Integrating RNA Sequencing (RNA‐Seq) data, we found that qVICs expressed higher levels of key genes related to chromatin remodeling including the acetyltransferase CREB binding protein (CREBBP). Next, we showed that inhibition of CREBBP prevented the transition of qVICs to aVICs. Consistent with this observation, we also observed reduced expression of CREBBP in AVS patient‐derived valve samples compared to valve samples from healthy donors, suggesting a role for histone modifiers in the progression of AVS.

## 2 RESULTS

### 2.1 Stiffness‐induced myofibroblast activation is associated with increased histone modifications and chromatin condensation

**FIGURE 1:**
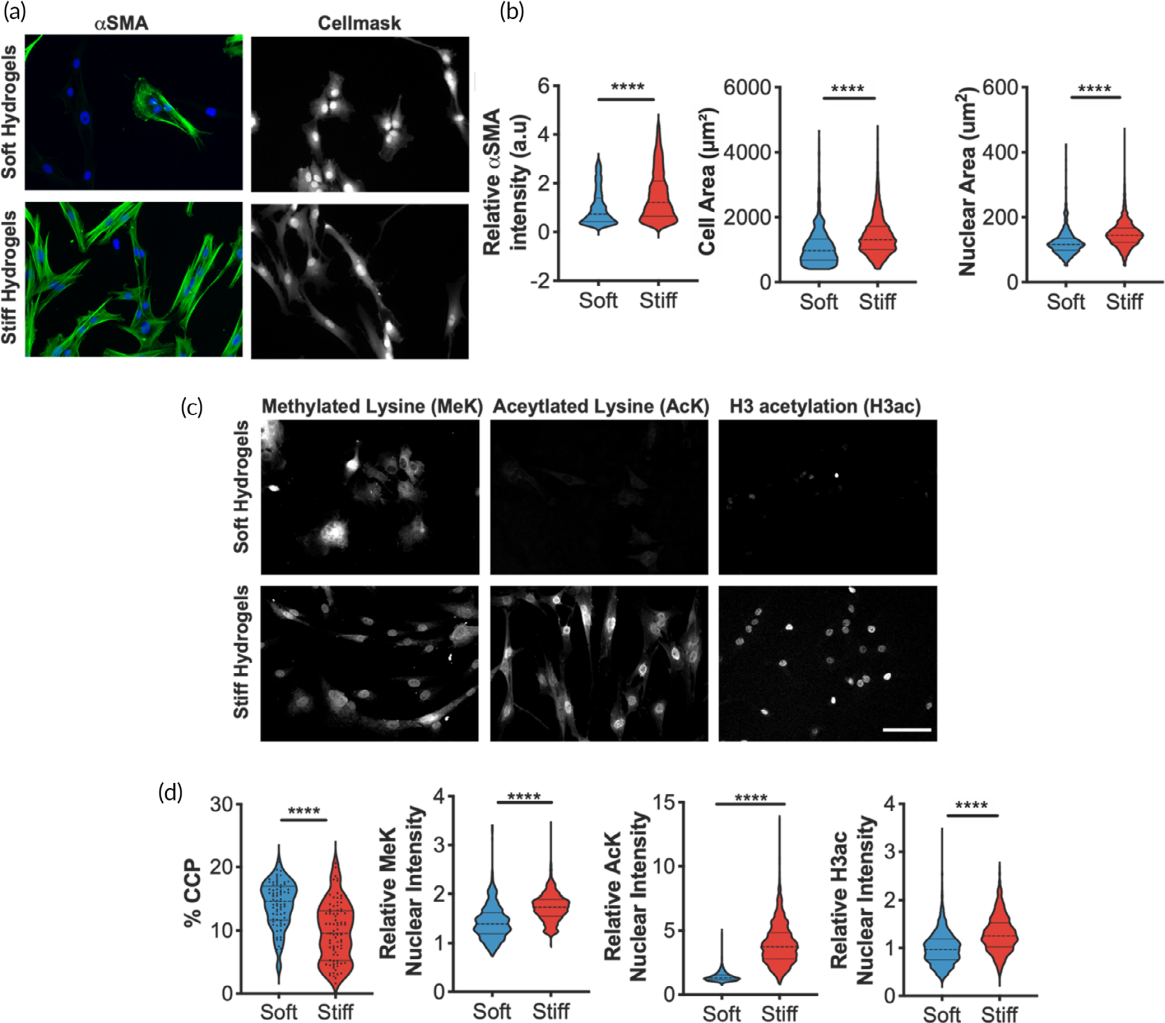
Histone modifications and chromatin condensation are altered when valve interstitial cells (VICs) are cultured on stiff or soft hydrogels. (a) Representative images of male VICs cultured on stiff or soft hydrogels when immunostained for αSMA (green) and DAPI (blue), Cellmask, scale bar = 100 um. (b) Quantification of myofibroblast‐like phenotypes using αSMA relative fluorescence intensity, cell area, and nuclear area for VICs on soft or stiff hydrogels. *n* > 592 cells. (c) Representative images of male VICs cultured on stiff or soft hydrogels when immunostained for methylated lysine (MeK), aceytlated lysine (AcK), and histone H3 acetylation (H3ac). Scale bar = 100 μm. (d) Quantification of chromatin condensation parameter (% CCP) for VICs cultured on stiff or soft hydrogels. *n* > 73 cells. Quantification of histone modifications (MeK, AcK, H3ac) for VICs cultured on stiff or soft hydrogels. *n* > 679 cells. All analyses were performed across >3 hydrogels. Wilcoxon signed rank test applied. **** *p* ≤ 0.001

PEG acrylate hydrogels^20^ were synthesized and used to study the role of microenvironmental stiffness on the activation of porcine‐derived VICs to myofibroblasts and their epigenetic remodeling in vitro. Soft hydrogel formulations were prepared with a Young's elastic modulus similar to healthy, porcine aortic tissue (E = ~5 kPa)^21^ (Figure S1A). Stiff hydrogels were designed with an elastic modulus to recapitulate the fibrotic valve tissue matrix, which can be 2–3× stiffer than healthy valves (E = ~13 kPa).^22^, ^23^ Porcine VICs were seeded onto these soft or stiff PEG hydrogels and cultured for 3 days prior to analysis. VICs maintained quiescence on soft hydrogels (qVICs) but exhibited an activated myofibroblast‐like phenotype on stiff hydrogels (aVICs), marked by expression of alpha smooth muscle actin (αSMA) fibers and increased cell area (Figure 1a,b).^13^, ^24^, ^25^, ^26^ Thus, as expected and shown previously,^15^ culture on soft hydrogels yields qVICs, while stiff hydrogels yield aVICs.

With this platform, we examined potential changes in nuclear morphology and chromatin architecture associated with transition to the aVIC phenotype. An increase in nuclear area was observed in VICs on stiff compared to soft hydrogels (Figure 1b). Since increased nuclear area is associated with chromatin remodeling,^27^ chromatin organization was next quantified by an automated image processing script.^28^ Using confocal microscopy to image the nuclei in high resolution, we calculated the chromatin condensation parameter (CCP) for qVICs and aVICs (Figure 1c). Reduced chromatin condensation was observed in aVICs compared qVICs, suggesting that the chromatin structure becomes more open during myofibroblast activation on stiff hydrogels (Figure 1d).

Having found that stiffness alters global chromatin condensation in VICs, histone modifications associated with changes in gene expression were examined (Figure 1d). By measuring the relative intensity of nuclear lysine methylation (MeK), lysine acetylation (AcK), and histone H3 acetylation (H3ac), it was found that all markers were significantly higher in aVICs compared to qVICs. These results indicate that the chromatin landscape is remodeled in response to matrix stiffness in VICs, generally toward a more open architecture. This finding led us to investigate the specific genomic regions that are epigenetically regulated by stiffness.

### 2.2 ATAC‐Seq identifies regions of increased chromatin accessibility in aVICs

**FIGURE 2:**
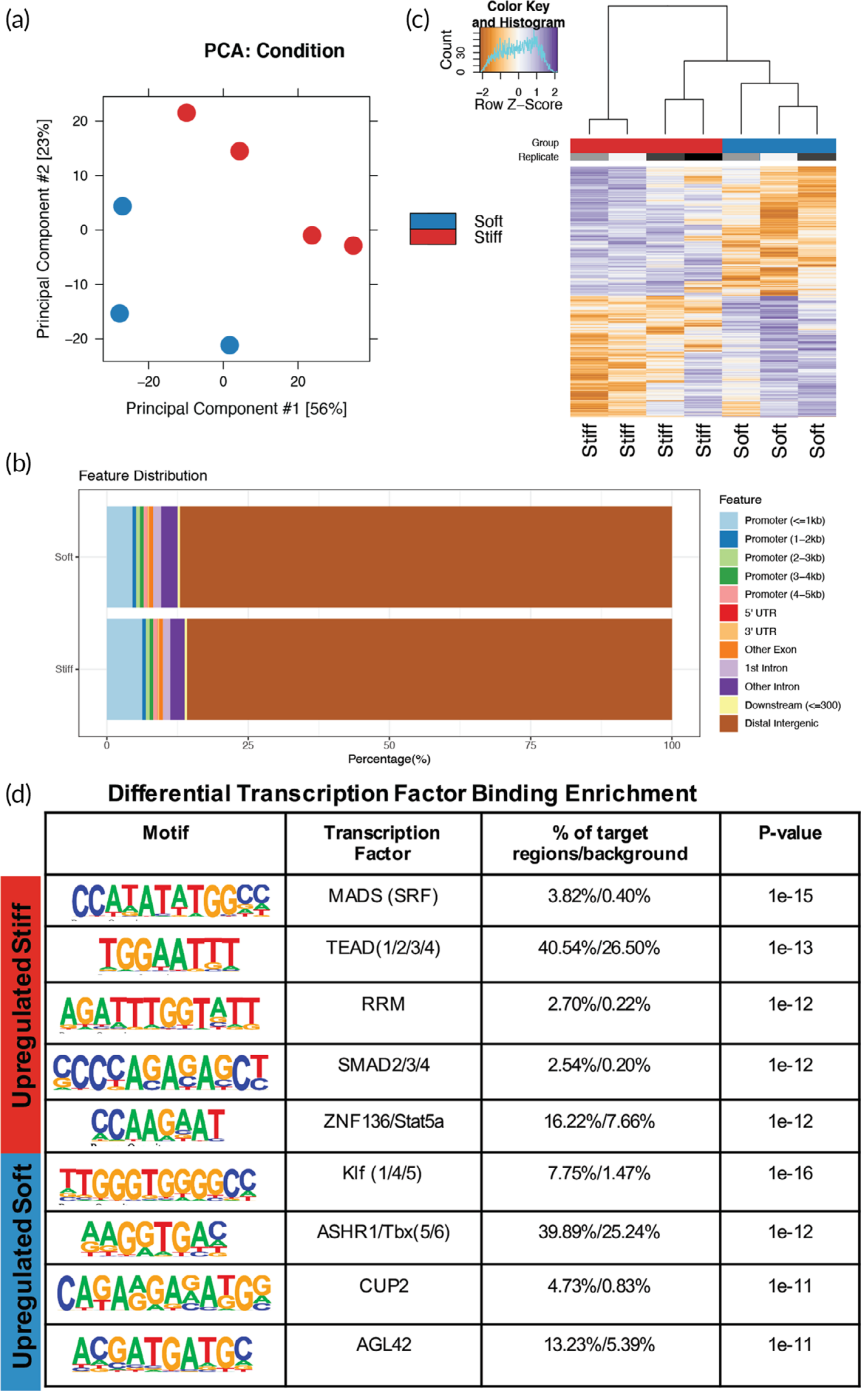
Chromatin accessibility differences in valve interstitial cells (VICs) cultured on stiff or soft hydrogels identified with ATAC‐sequencing. (a) Principal component analysis (PCA) of the ATAC‐seq data sets. (b) Annotation of the location of ATAC peaks in stiff or soft samples to the UCSC susScr11 reference genes. (c) Heatmap and clustering of ATAC‐seq differentially accessible peaks. (d) Motif enrichment analysis of differentially accessible peaks in stiff or soft samples using Homer de novo motifs with Homer's motif library

ATAC‐Seq was performed on qVICs on soft hydrogels and aVICs on stiff hydrogels to identify the open chromatin regions as a function of substrate stiffness. Since we aimed to understand the role of matrix mechanical cues on chromatin remodeling, we modified the Omni‐ATAC‐Seq protocol to preserve cell–matrix interactions.^29^ Principal component analysis (PCA) of all the accessible regions indicated that independent replicates for VICs cultured on soft or stiff hydrogels were clustered together (Figure 2a). There was a progressive shift across both PC1 and PC2 axes that correlated with myofibroblast activation. In general, the genomic distribution of ATAC peaks across the stiff and soft datasets were similar (Figure 2b). The majority of peaks were annotated to distal intergenic regions, which may represent enhancers.^30^ Differential analysis of ATAC‐Seq peaks revealed VICs on stiff hydrogels had more open regions compared to VICs on soft hydrogels (907 vs. 768) (Figure 2c). In addition to the CCP and histone modification results, the ATAC‐Seq data support the hypothesis that stiffness controls chromatin accessibility in VICs.

To identify candidate transcription factors that may be binding to the differentially accessible sites, we performed de novo and known motif analysis using Hypergeometric Optimization of Motif EnRichment (HOMER)^31^ on the regions that were more accessible in VICs cultured on stiff compared soft hydrogels and vice versa (Figure 2d, Tables S1 and S2). Among the enriched transcription factor motifs with increased accessibility on stiff hydrogels were transcription factors previously associated with mechanosensing, including TEA domain family members (TEADs) and serum response factor (SRF). TEADs bind to mechanosensitive nuclear YAP to promote fibrotic gene expression in VICs.^32^ We next tested whether YAP‐TEAD association was required for chromatin remodeling in VICs on stiff hydrogels. Like prior reports, VICs on stiff hydrogels had more nuclear YAP than cytoplasmic, when compared to VICs on soft hydrogels (Figure S1B). When YAP‐TEAD association was inhibited with verteporfin (1 μm) on stiff hydrogels, VIC nuclear H3ac expression was reduced (Figure S1C). This result indicates that YAP‐TEAD is important for stiffness‐induced aVIC chromatin remodeling.

Open regions in qVICs on soft hydrogels also had enriched motifs for transcription factors, including Krüppel‐like factors (KLFs) and TBX5/6 (T‐box) (Figure 2d), suggesting that these transcription factors may promote expression of qVIC genes. Additionally, we used transcription factor enrichment analysis (TFEA)^33^ on the ATAC‐seq datasets for further characterization and found enrichment of similar transcription factors identified using HOMER, like KLFs and TEADs (Table S3). Using HOMER on all ATAC peaks from VICs cultured on stiff and soft hydrogels, we observed significant enrichment of motifs for cell proliferation and chromatin remodeling, including Fos, Fra1, BATF, Atf3, JunB, CTCF, and AP‐1 (Table S4 and S5). Since these transcription factors were identified in both qVICs and aVICs, they may be playing a role in defining the VIC cell type (as opposed to an endothelial cell, e.g.). Overall, these findings highlight the complexity of the VIC phenotype but also indicate transcription factors that may control the VIC response to different matrix mechanical cues.

### 2.3 Integration of ATAC‐Seq and RNA‐Seq identifies key genes in stiffness‐induced VIC activation

**FIGURE 3:**
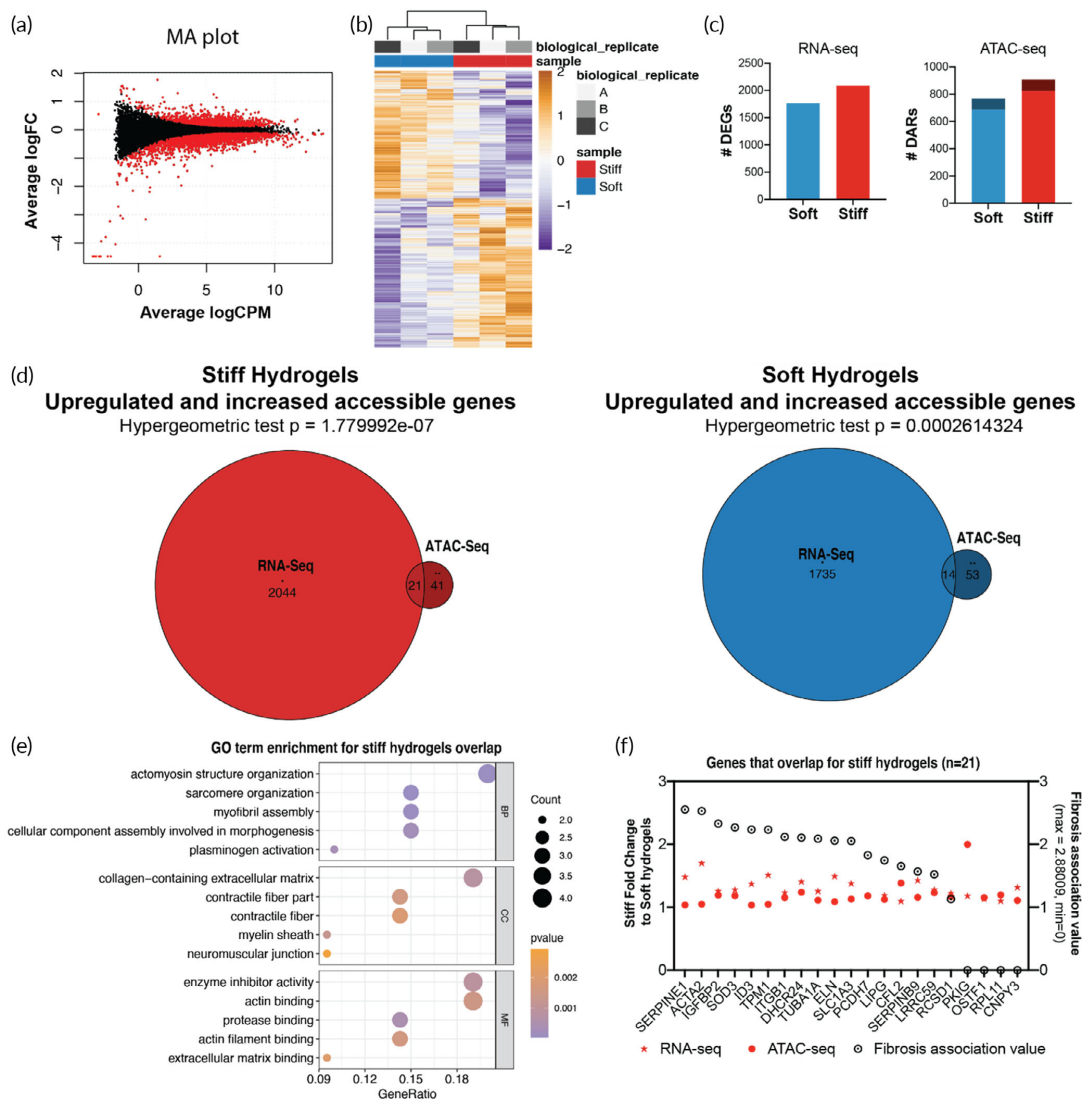
Integration of ATAC‐seq and RNA‐seq in quiescent valve interstitial cells (qVICs) and activate to myofibroblasts (aVICs). (a) MA plot of differentially expressed genes found between valve interstitial cells (VICs) cultured on stiff and soft hydrogels where red dots indicate genes found to be significantly different (false discovery rate [FDR] < 0.05). (b) Heatmap and clustering of RNA‐seq differentially expressed genes. (c) Number of differentially expressed genes (DEGs) found using RNA‐seq and the number of differentially accessible regions found using ATAC‐seg. Darker colors in ATAC‐Seq graph indicate differentially accessible regions (DARs) that annotate to genes and lighter colors indicate DARs that annotate to distal intergenic regions according to ChIPseeker. (d) Overlap between genes that are upregulated and more accessible in VICs cultured on stiff or soft hydrogels. (e) Gene ontology (GO) term analysis of the gene‐set in the overlap between RNA‐seq and ATAC‐seq datasets from stiff hydrogel samples. *p* < 0.10. (f) Fold change of genes upregulated and more accessible from RNA‐Seq and ATAC‐Seq on stiff hydrogels relative to soft hydrogels in descending order of fibrosis association value

To elucidate how changes in open chromatin regions from the ATAC‐Seq analysis correlate with gene expression changes in aVICs, RNA‐Seq was performed and integrated with the ATAC‐Seq dataset. RNA‐Seq was performed on three independent replicates of qVICs from soft hydrogels and aVICs from stiff hydrogels. Based on the differential expression analysis (false discovery rate [FDR] < 0.05), there were 1763 upregulated genes on soft hydrogels and 2086 upregulated genes on stiff hydrogels (Figure 3a–c). To integrate these datasets, differentially accessible regions identified by ATAC‐Seq were annotated with the closest gene and a genomic feature (i.e., promoter, UTR, etc.) (Figures 2b and 3c). Differentially accessible regions in distal intergenic locations were excluded since there is low confidence that these regions would regulate the closest gene. After the exclusion of distal intergenic regions, there were 81 and 83 open regions annotated to genes in the soft sample and stiff sample datasets, respectively.

We found several upregulated genes from the RNA‐Seq that significantly overlapped with the genes with increased accessibility from the ATAC‐Seq (Figure 3d). Specifically, 21 genes that were identified overlapped between the RNA‐Seq and ATAC‐Seq datasets for the stiff samples, and 14 genes overlapped for the soft samples. When gene ontology (GO) term analysis was performed on the overlapped gene set from the stiff samples, terms like “actomyosin structure organization” and “collagen‐containing extracellular matrix” were enriched (Figure 3e). In contrast, genes overlapping in soft samples were enriched for terms like “cell‐cell junction assembly” and “phospholipid binding” (Figure S2A). To investigate the relevance of the individual genes that overlapped in the ATAC‐Seq and RNA‐Seq datasets to the VIC myofibroblast phenotype, the strength of the functional association of each gene to the fibrosis gene set^34^ was reported (Figure 3f, Figure S2B). The genes identified in the stiff samples associated with fibrosis more than the soft samples (Figure S2C), like actin alpha 2, smooth muscle (ACTA2) and plasminogen activator inhibitor 1 (SERPINE1). A complete list of the differentially accessible and expressed genes and their overlap can be found in the Supplementary Information (Tables S5–S10). Taken together, these results suggest that increased stiffness regulates chromatin accessibility to encode fibrotic, myofibroblast‐like programming in VICs.

### 2.4 Soft hydrogels upregulate expression of chromatin remodeling genes in VICs

**FIGURE 4:**
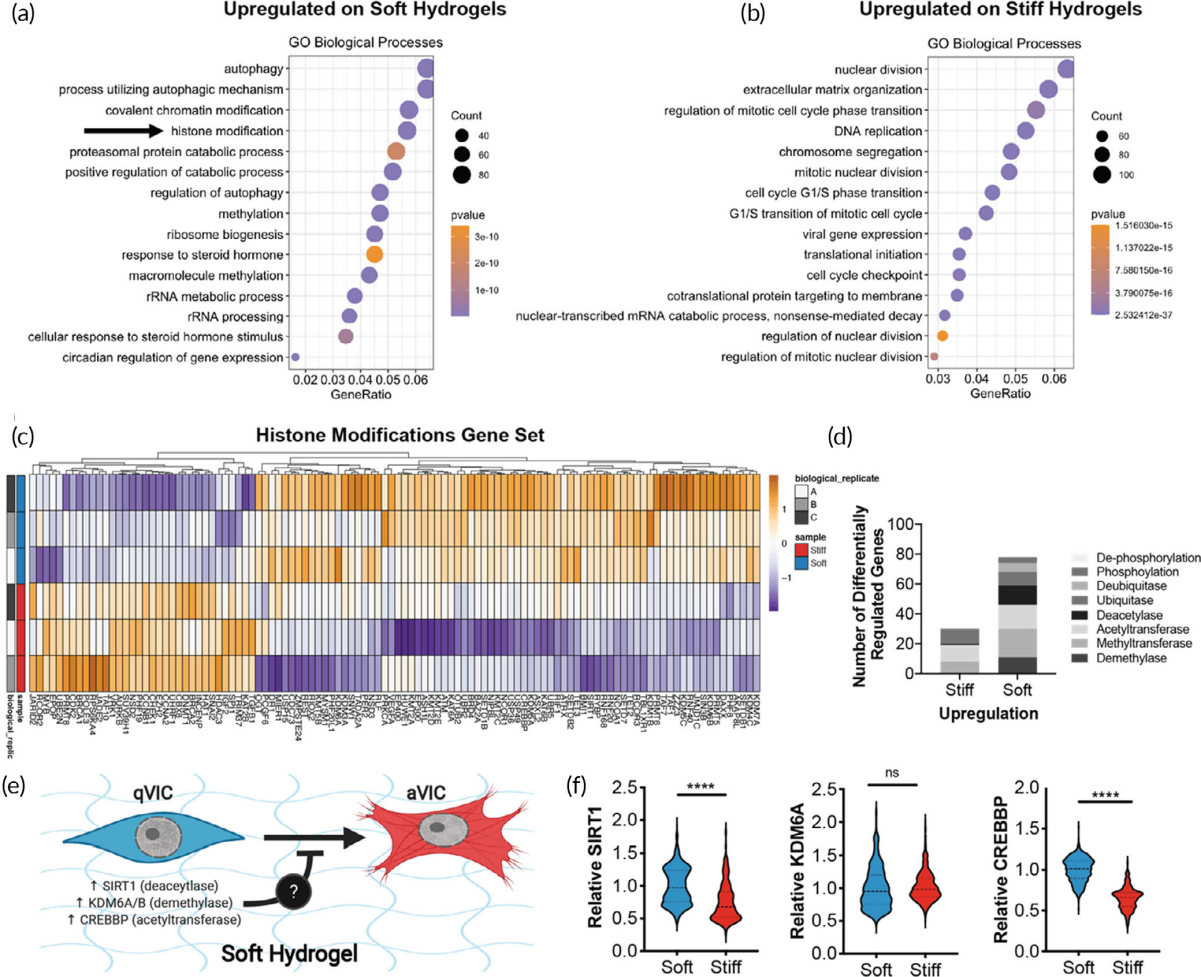
Soft hydrogels upregulate genes related to chromatin remodeling. (a,b) Gene ontology (GO) biological processes term enrichment of genes upregulated in valve interstitial cells (VICs) on soft (a) or stiff (b) hydrogels. (c) Heatmap of differentially expressed genes within the GO term category of histone modifications. (d) Number of differentially regulated genes related to chromatin remodeling in VICs cultured on stiff or soft hydrogels. (e) Illustration of hypothesis that upregulation of chromatin remodelers (e.g., SIRT1, KDM6A/B, or CREBBP) in VICs on soft hydrogels protects against unwanted myofibroblast activation. Created using Biorender.com. (f) Quantification of protein levels of SIRT1, KDM6A, and CREBBP in VICs cultured on soft or stiff hydrogels. *n* > 403 cells from three hydrogels. Unpaired two‐way student's *t*‐test applied. **** *p* ≤ 0.001, *p* ≤ 0.005

We next investigated the chromatin remodelers that control chromatin accessibility, specifically in response to stiffness, by performing a deeper analysis of the transcriptome dataset. According to GO term annotation of the differentially expressed genes, biological processes related to cell cycle progression and extracellular matrix organization were upregulated on stiff hydrogels (Figure 4a). To test the biological relevance of these GO terms, we tested VIC proliferation on soft and stiff hydrogels. We found that VICs proliferate more on stiff hydrogels compared to soft hydrogels (Figure S3). In contrast, the biological processes upregulated on soft hydrogels were related to autophagy, metabolic processes, and histone modifications (Figure 4b). Visualization of the expression levels of the genes associated with the histone modification GO term (0016570) highlights that the majority of genes associated with histone modifications are upregulated in VICs on soft hydrogels compared to stiff hydrogels (Figure 4c). Further, VICs on soft hydrogels had increased histone modifier activity based on functional annotation of the histone modification gene set (Figure 4d).

Based on this observation, we hypothesized that upregulated chromatin remodeling on soft hydrogels inhibits VIC myofibroblast activation (Figure 4e). Further, we hypothesized that increased expression of a specific chromatin remodeler may play a role in preserving VIC quiescence. We interrogated three genes that were upregulated in the transcriptomic datasets of the soft hydrogel samples: (1) SIRT1 (Sirtuin 1), a histone deacetylase that is downregulated in valves from AVS patients compared to control,^7^ (2) KDM6A/B (lysine demethylase 6A and B), which are H3K27me3‐specific histone demethylases that protect against renal fibrosis,^35^ and (3) CREBBP (CREB‐binding protein), an acetyltransferase that protects against VIC calcification.^36^ Both SIRT1 and CREBBP were upregulated in VICs cultured on soft hydrogels compared to stiff hydrogels (Figure 4f, Figure S4) based on quantification of immunostained cells. These data suggest that qVICs on soft hydrogels may have increased chromatin modifier activity and that may play a role in maintaining quiescence on soft hydrogels.

### 2.5 CREBBP protects qVICs from myofibroblast activation on soft matrices and recovers the qVIC phenotype on stiff matrices

**FIGURE 5:**
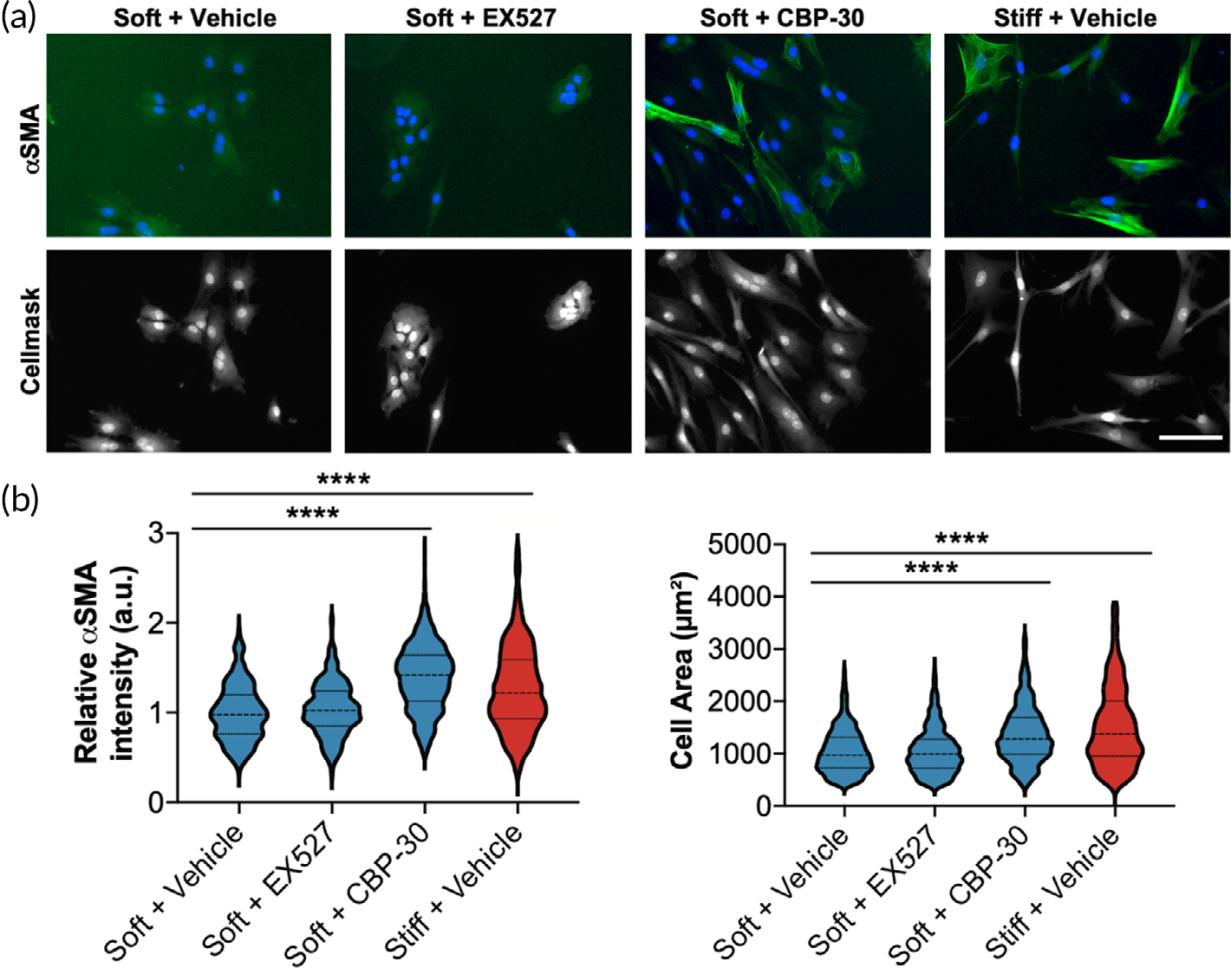
CREB binding protein (CREBBP) inhibition increases myofibroblast activation of soft hydrogels. (a) Representative images of valve interstitial cells (VICs) treated with vehicle, 50 μM EX527 (SIRT1 inhibitor), or 10 μM CBP‐30 (CREBBP inhibitor). αSMA = green, DAPI = blue. Cellmask = gray. Scale bar = 100 μm. (b) Quantification of relative cellular αSMA intensity and cell area with drug treatments. *n* > 357 cells from >4 hydrogels. One‐way analysis of variance (ANOVA) with Bonferroni's multiple comparison test applied. ****p < 0.0001

Next, we investigated a mechanistic link between SIRT1 or CREBBP in maintaining the qVIC phenotype from myofibroblast activation on soft hydrogels. SIRT1 and CREBBP were inhibited using established small molecule inhibitors, EX527 and SG‐CBP‐30 (CBP‐30), at previously determined concentrations (Figure S5).^37^, ^38^ VICs were cultured on soft hydrogels and treated with vehicle (DMSO), EX527 (50 μM), or CBP‐30 (10 μM) for 3 days, then quantified myofibroblast activation using αSMA expression and cell area as readouts. Inhibition of CREBBP with CBP‐30 increased αSMA expression and cell area, while EX527 SIRT1 inhibition did not (Figure 5a,b). This result indicates that CREBBP expression in qVICs inhibits myofibroblast activation under soft, physiological conditions.

**FIGURE 6:**
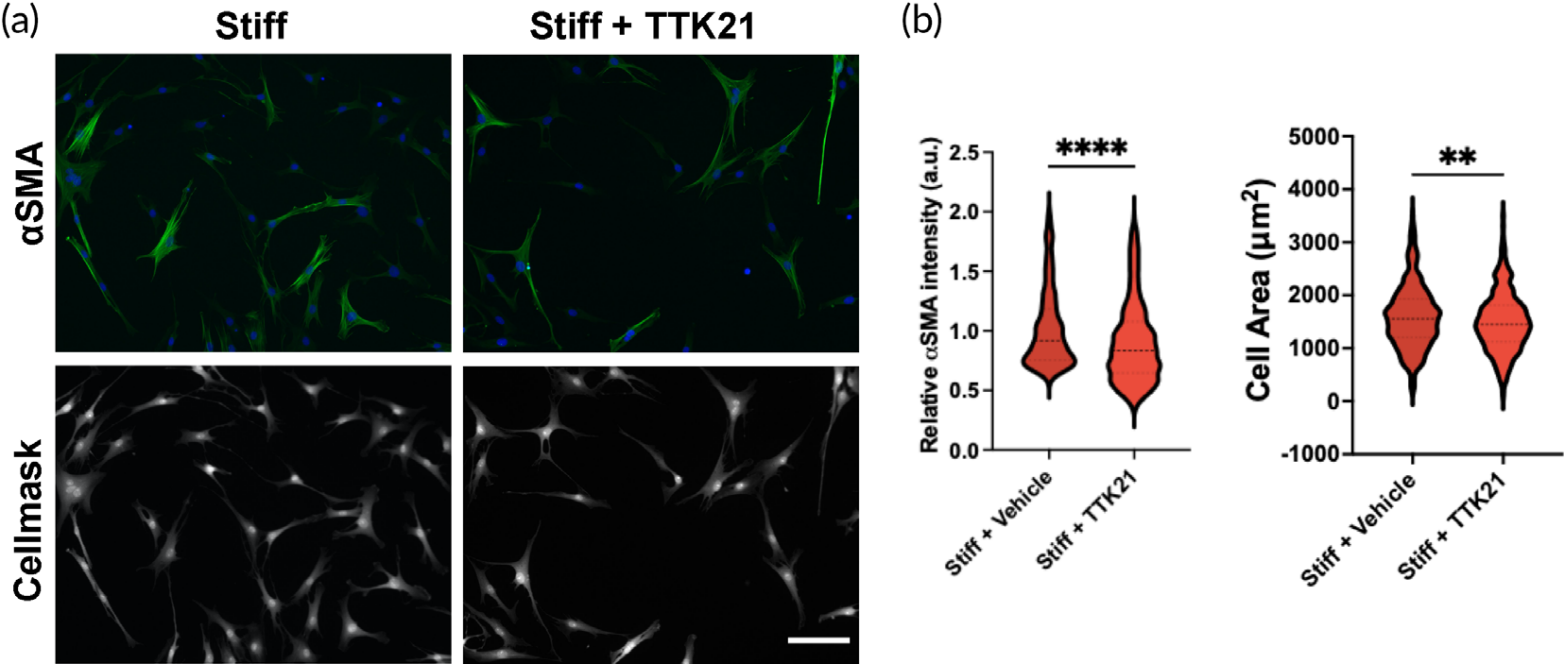
CREB binding protein (CREBBP) activator decreases myofibroblast activation of stiff hydrogels. (a) Representative images of valve interstitial cells (VICs) treated with vehicle, 10 μM TTK21 (CREBBP activator). aSMA = green, DAPI = blue. Cellmask = gray. Scale bar = 100 μm. (b) Quantification of relative cellular aSMA intensity and cell area with drug treatments. *n* > 427 cells from >4 hydrogels. One‐way analysis of variance (ANOVA) with Bonferroni's multiple comparison test applied. **p < 0.01, ****p < 0.0001

Based on this observation, we investigated whether activating CREBBP on stiff matrices would promote the qVIC phenotype cultured in this microenvironment. CREBBP was activated using a small molecule activator, TTK21 at a concentration determined previously (Figure S6).^39^, ^40^, ^41^ VICs were cultured on stiff hydrogels and treated with vehicle (DMSO) or TTK21 (10 μM) for 3 days. Myofibroblast activation was quantified using αSMA expression and cell area using Cellmask staining. Activation of CREBBP with TTK21 decreased both αSMA expression and cell area (Figure 6a,b). These data suggest that CREBBP has a protective role against myofibroblast activation under stiff conditions.

**FIGURE 7:**
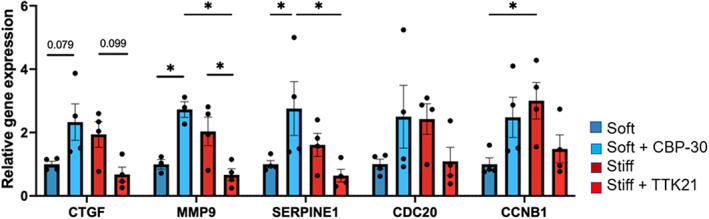
CREB binding protein (CREBBP) inhibitor increases and CREBBP activator decreases mRNA levels of myofibroblast and proliferative genes. mRNA levels of myofibroblast and proliferative genes on soft hydrogels treated with vehicle and 10 μM CBP‐30 (CREBBP inhibitor) and on stiff hydrogels treated with vehicle and 10 μM TTK21 (CREBBP activator). One‐way analysis of variance (ANOVA) with Bonferroni's multiple comparison test applied. *p < 0.05

Next, we investigated whether CREBBP inhibition or activation would cause a change in expression of genes associated with myofibroblast activation and cell proliferation. VICs were cultured on soft and stiff hydrogels and treated with vehicle (DMSO), CREBBP inhibitor (CBP‐30, 10 μM, on soft hydrogels), or CREBBP activator (TTK21, 10 μM, on stiff hydrogels) for 3 days. Expression of myofibroblast‐associated genes (CTGF, MMP9, and SERPINE1) and proliferation‐associated genes (CDC20, CCNB1) was quantified with qPCR (Figure 7). On soft hydrogels, both myofibroblast‐ and proliferation‐associated genes increased upon CREBBP inhibition. On stiff hydrogels, myofibroblast and proliferation associated genes decreased upon CREBBP activation. These results suggest that CREBBP plays an active role in promoting a qVIC phenotype.

We next investigated the role of CREBBP on cell proliferation since we found that proliferation correlates with myofibroblast activation (Figure S4). We cultured VICs on soft hydrogels while treated with vehicle or 10 μM CBP‐30 to deactivate CREBBP and on stiff hydrogels while treated with vehicle for 3 days. We then pulsed cells with 5‐ethynyl‐2′‐deoxyuridine (EdU) for 24 h and quantified proliferation by the presence of EdU in cell nuclei. VICs had higher levels of proliferation on soft hydrogels when with CREBBP inhibition (Figure S7). Overall, these results suggest that CREBBP promotes the qVIC phenotype on soft hydrogels by actively suppressing activation and proliferation.

### 2.6 CREBBP is negatively correlated with aortic valve disease

**FIGURE 8:**
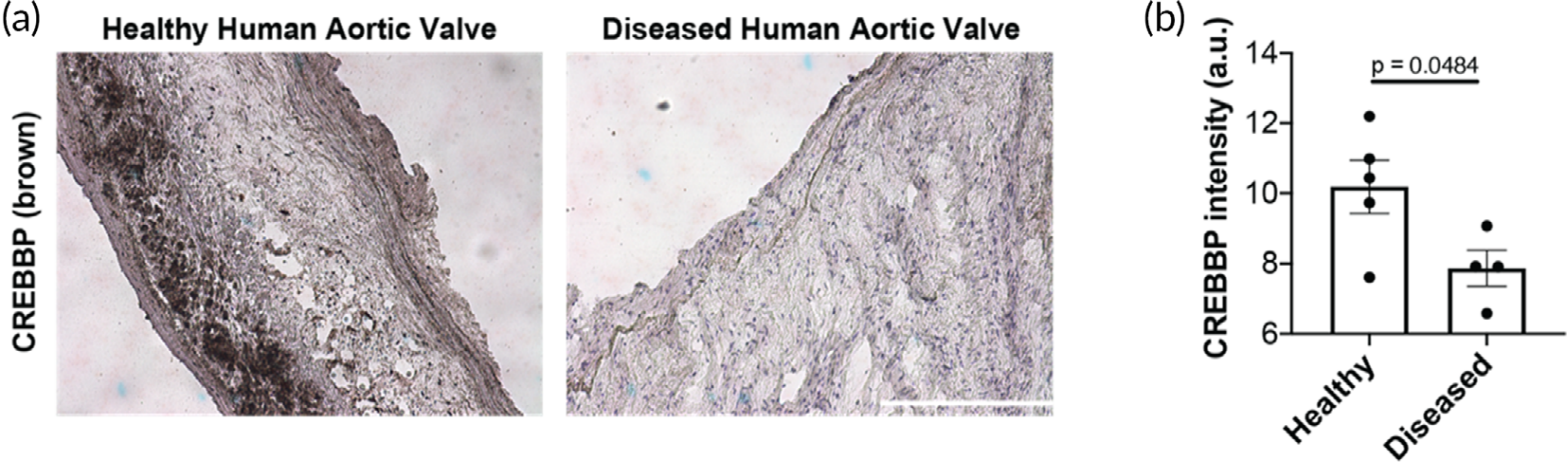
CREB binding protein (CREBBP) expression is higher in normal human aortic valves compared to diseased valves. (a) Representative images of stained tissue sections from healthy or diseased human aortic valve samples (see supplement for de‐identified patient information). Hematoxylin in purple. CREBBP in brown. Scale bar = 200 μm. (b) Quantification of CREBBP levels in healthy (*n* = 5) or diseased (*n* = 4) human valve samples. Unpaired two‐way Student's *t*‐test applied. Data ± SEM

Notably, expression levels of CREBBP are reduced in valve leaflets from patients with aortic valve disease (*n* = 4) compared to healthy patient leaflets (*n* = 5) (Figure 8a,b, Table S11). Taken together, these results indicate that CREBBP expression in VICs on soft hydrogels prevents myofibroblast activation and may play a protective role in maintaining valve quiescence in healthy human valves.

## 3 DISCUSSION

We demonstrate that stiffness‐induced myofibroblast activation is associated with chromatin remodeling, which regulates expression of pro‐fibrotic genes. By combining next generation sequencing techniques, we found that the chromatin landscape enables greater pro‐fibrotic gene expression in aVICs, compared to qVICs. We identify differential motif enrichment for transcription factor binding sites in aVICs compared to qVICs, indicating differential regulation of VIC chromatin accessibility. Further, transcriptomics analysis revealed that qVICs on soft hydrogels have increased gene expression of chromatin remodelers, which protects qVICs from myofibroblast activation. Specifically, we found that CREBBP, a histone acetyltransferase, plays a role in maintaining the qVIC phenotype. Notably, CREBBP is downregulated in diseased human valve samples compared to control valves, suggesting loss of expression is associated with AVS progression through VIC myofibroblast activation.

Importantly, biophysical cues, such as increased matrix stiffness, can control cellular phenotype via epigenetic remodeling in many cell types.^42^, ^43^ For example, in a 3D model of breast cancer, a stiff ECM induced a tumorigenic phenotype in breast epithelial cells through changes in chromatin state.^44^ Additionally, increased matrix stiffness increased global chromatin accessibility in lung fibroblasts.^45^ Here, we show that VICs on stiff synthetic ECM mimics remodel their chromatin to access and increase expression of myofibroblast‐like genes, including ACTA2 (αSMA) and SERPINE1 (PAI‐1), suggesting that control of myofibroblast activation by stiffness associates with chromatin remodeling. However, there are some limitations to the interpretation of these results, namely as causes or effects of VIC activation. It is likely that some of the readouts identified act downstream of VIC activation, rather than upstream of VIC activation. Further investigations into chromatin accessibility, gene expression differences, and VIC activation using inhibitors and knockdown studies would help address these limitations more conclusively. Beyond matrix signaling, previous studies have also demonstrated that osteogenic media can influence the VIC epigenetic landscape.^10^, ^18^, ^36^, ^46^ Interestingly, matrix stiffness and cytokine treatment have significantly different effects on myocardial fibroblast secretomes,^47^ suggesting that perhaps the mode of VIC activation may also have distinctive effects on epigenetic profiles. Further investigations are needed to identify the differential effect of activation cues on VIC chromatin accessibility and gene expression. One recent study suggests that myofibroblast activation through increased matrix stiffness encodes pro‐fibrotic gene expression via chromatin accessibility in lung fibroblasts, indicating this remodeling may be a conserved mechanism for myofibroblast activation in cells throughout the body.^45^ Since valves from AVS patients are stiffer than those in healthy individuals,^23^ these findings imply that the valve ECM may play a role in AVS‐mediated epigenetic changes, and vice versa, propagating a vicious cycle. Interestingly, nuclear mechanosensing was found to mediate stiffness‐induced chromatin remodeling in bone marrow derived human mesenchymal stem cells (hMSCs)^27^; however, this is likely not the case in VICs. We recently demonstrated that VICs activate to myofibroblasts on stiff hydrogels even when nuclear mechanosensing is disrupted.^19^ It is possible that in VICs, traditional signaling controls chromatin remodeling through mechanotransduction mechanisms (i.e., YAP/TAZ, SRF, SMADs).^48^

In the results reported herein, we found that loss of CREBBP activity resulted in transformation of qVICs to aVICs in the absence of stiff mechanical cues. Similar to these findings, CREBBP protein levels have been negatively correlated with calcification in patient valves.^36^ Thus, CREBBP upregulation on soft hydrogels may preserve the qVIC phenotype. Interestingly, CRISPR‐induced upregulation of CREBBP in VICs cultured in osteogenic media prevented the obVIC phenotype.^36^ Maintenance of the qVIC phenotype by CREBBP could occur through several mechanisms since CREBBP acetylates both histone and nonhistone proteins.^49^ We found that CREBBP inhibition increased H3ac levels on soft hydrogels, similar to levels observed on stiff hydrogels and contrary to its role as a histone modifier. CREBBP can also acetylate other proteins to regulate their activity, like YAP,^50^ or cytoskeletal components to control their formation and stability.^51^ These findings suggest that CREBBP activation could be utilized as an AVS therapy to reduce aVIC and obVIC phenotypes. However, it is important to note that a recent study found that CREBBP promotes the pro‐fibrotic phenotype in idiopathic pulmonary fibrosis and Dupuytren's disease,^52^ highlighting the concept that the CREBBP‐based mechanism presented here may apply specifically to VIC biology, rather than fibroblasts in general.

CREBBP likely operates in concert with other mediators to control the aVIC and qVIC phenotypes. Serum response factor (SRF) binding motifs were enriched in regions that were more accessible in VICs on stiff hydrogels versus. soft hydrogels. Mechanical stress can cause nuclear translocation of MRTFA, which associates with SRF to activate the transcription of pro‐fibrotic genes.^53^ Interestingly, TEADs were also found to be enriched in VICs on stiff hydrogels. TEADs associate with YAP/TAZ in the nucleus to promote transcription in response to mechanical inputs^54^; however, the role of this mechanism in VIC phenotypes and chromatin remodeling is unclear. Previous work has shown that nuclear‐localization of YAP increases with increased matrix stiffness in VICs, but its inhibition reduces αSMA expression.^22^, ^55^ In the present study, we show that YAP inhibition also reduces histone acetylation, indicating YAP‐TEAD mediated mechanotransduction is important for the aVIC chromatin state. Intriguingly, ATAC‐Seq analysis also found transcription factors enriched in VICs on soft hydrogels, including KLFs. KLF4 is downregulated in patients with bicuspid valves (BAV), which develop significant calcification compared to patients with tricuspid aortic valves (TAV).^56^ While there are no reports indicating KLFs in maintaining qVIC phenotypes, it is notable that KLF4 is used to induce pluripotency in other cell types.^57^ Since qVICs can transform into aVICs or obVICs, perhaps KLF maintains the plasticity of qVIC phenotype. KLF3 activity has also been previously shown to recruit CREBBP to active enhancer sites. KLF3 and CREBBP have been reported to work together in regulating cell cycle and proliferation related genes including CCNB1.^58^ It is possible that KLFs may also be playing an upstream role in regulating CREBBP activity in a mechanosensitive manner. Future investigations are needed to understand the qVIC phenotype and the transcription factors controlling its state.

More generally, this work provides a wealth of information that could contribute to the field's understand of VIC biology. While we chose to focus on three potential candidates (SIRT1, KDM6A/B, and CREBBP) and their role in maintaining qVIC phenotype, it is likely that other chromatin remodeling proteins regulate VIC phenotype. These datasets and their mining could thus serve as bases for future studies. For example, the ATAC‐Seq dataset identified transcription factor binding motifs enriched in VICs across stiff and soft hydrogels (Table S4 and S5) may be useful for developing iPSC differentiation protocols. Currently, there is only a single iPSC‐derived VIC protocol available, and it relies on 3D encapsulation of cells.^59^ In addition, the datasets presented here should allow for investigation of other mechanisms responsible aVIC transformation that may be implicated in AVS progression.

## 4 CONCLUSIONS

In conclusion, we identify stiffness‐dependent global changes in chromatin accessibility and transcription as VICs transition from a quiescent to activated phenotype. The integration of ATAC‐Seq and RNA‐Seq identified that the chromatin of aVICs on stiff hydrogels instilled pro‐fibrotic gene expression. Moreover, upregulation of acetyltransferase CREBBP prevented VIC activation. These findings emphasize the fundamental role of chromatin remodeling and epigenetics in VIC response to biophysical cues. Although we characterize un‐activated VICs as “quiescent,” we demonstrate, perhaps for the first time, that active epigenetic processes are required to maintain VIC phenotypic quiescence, under the conditions we studied. We propose the term “biologically vigilant quiescence” to describe those processes.

## 5 MATERIALS AND METHODS

### 5.1 Synthesis of hydrogel components and characterization of properties

PEGdiPDA was synthesized and characterized as previously described^20^; >90% functionality was verified by ^1^H NMR. The adhesive peptide acrylamide diethylene glycol‐diethylene glycol‐glycine‐arginine‐glycine‐aspartic acid‐serine‐glycine (Ac‐OOGRGDSG) was synthesized via 9‐fluorenylmethyloxycarbonyl (Fmoc)/1‐[bis(dimethylamino)methylene]‐1H‐1,2,3‐triazolo[4,5‐b] pyridinium 3‐oxid hexafluorophosphate (HATU) coupling strategy on RINK Amide MBHA resin (0.500 mmol) using a Protein Technologies Tribute automated peptide synthesis machine and characterized using electrospray ionization (ESI) mass spectroscopy as previously described.^20^

A shear rheometer (TA Instruments DH‐R3) was used to measure the modulus of the PEGdiPDA hydrogel before and after degradation. In brief, optically thin PEGdiPDA hydrogels (thickness = 50 μm) were polymerized in situ between 8‐mm diameter parallel plates. Hydrogel evolution was monitored using a dynamic time sweep (*γ* = 1%; *ω* = 1 rad/s; determined to be in the linear regime for this material) until the storage modulus (*G*′) reached a plateau (XX = min). For in situ degradation, a quartz plate was used and the rheometer was equipped with a 365 nm light source (λ = 365 nm; I_0_ = 10 mW/cm^2^; Omnicure 1000, LumenDynamics). After network formation, the hydrogel was exposed to 365 nm light for 6 min and the change in storage module was monitored using the same dynamic time sweep parameters. The same dynamic time sweep measurements were used to obtain the storage modulus. The shear modulus was measured and converted to Young's modulus (*E*) with 0.5 Poisson's ratio.

### 5.2 Hydrogel formation

The preparation of PEGdiPDA, photo‐softening hydrogels was adapted from previously described protocols 20. PEGdiPDA was co‐polymerized with poly(ethylene glycol) monoacrylate (PEGA; Mn ∼400 Da; Monomer‐Polymer and Dajac Laboratories) and acryl‐OOGRGDSG in PBS via redox‐initiated free‐radical polymerization. Gel solutions were prepared with 7.0 wt% PEGdiPDA, 6.8 wt% PEGA, 5 mM acryl‐OOGRGDSG, 0.2 M ammonium persulfate, and 0.1 M tetramethylethylenediamine (TEMED). Gels were formed on acrylated cover glass with a diameter of 12, 15, or 25 mm and a thickness of 100 μm and allowed to cure for 6 min before placing in PBS. Gels were rinsed in PBS before cell seeding. Soft hydrogels were prepared by irradiating the initial photo‐softening hydrogels with UV light (*λ* = 365 nm; *I*_0_ = 10 mW/cm^2^) for 6 min.

### 5.3 VIC isolation and culture

Porcine VICs were isolated as previously described^60^ from both male and female pigs. Briefly, porcine hearts were shipped overnight from Hormel (Minnesota). The aortic valve leaflets from >10 animals were dissected and placed in warmed wash buffer (Earle's salt solution, E2888). Wash buffer was aspirated, and leaflets were incubated at 37C, 5% CO_2_ while shaking with 1 mg/mL collagenase II (Worthington Biochemical LS004177) solution for 1 h to remove the endothelial layer. The supernatant was aspirated, and fresh 1 mg/mL collagenase II solution was added. Leaflets were incubated for 2 h, and vortexed for 1 min to break‐up the tissue. The cell solution was filtered through 100 μm filter and centrifuged for 30 min at 1000 rpm. The cell pellet was suspended in 15% FBS M199, 1% penicillin–streptomycin, and 0.5 μg/ml fungizone and grown until 90% confluence at 37°C, 5% CO_2_ after which they were frozen down. VIC cryovials were added to 1% FBS M199 media and seeded onto hydrogels at 20,000 cells/cm^2^, unless otherwise specified. Media was changed every other day. Male VICs were used for all studies with the exception of the RNA‐Seq datasets where a mixed population of male and female VICs was used. For inhibitor studies, cells were seeded onto hydrogels in the presence of verteporfin (Sigma SML0534), EX527 (Cayman Chemicals 10009798), or SGC‐CBP30 (Cayman Chemicals 14,469) diluted in DMSO (Sigma D2650) at a maximum concentration of 1 μl/ml.

### 5.4 Immunostaining, imaging, and analysis

Cells were fixed in 4% paraformaldehyde (Electron Microscopy Sciences 15710) for 20 min and permeabilized in 0.01% Triton X100 for 1 h at room temperature. Cells were blocked in 5% BSA (Sigma BP1600) for 1 h, and primary antibodies added and incubated at 4°C overnight. Primary antibodies used are as follows in 5% BSA: aSMA (abcam ab7817 1:1000), YAP (Santa Cruz Biotechnology sc‐101199, 1:500), Methylated Lysine (Novus Biologics NB600‐824, 1:500), Acetylated Lysine (abcam ab190479, 1:300), Histone H3 acetylation (EMD Millapore 06‐599, 1:1000), and CREBBP (abcam ab2832, 1:1000). Cells were washed, and secondary antibodies were added for 30 min. Cells were imaged with automated Perkin Elmer Operetta. Cell area, nuclear area, antibody intensity was quantified using Perkin Elmer's Harmony software. For αSMA measurements, values were normalized to Cellmask intensity.

### 5.5 Chromatin condensation imaging and analysis

Immunostained nuclei were imaged and analyzed for chromatin condensation parameter (CCP) as previously described.^28^ Briefly, nuclei were imaged with a laser scanning confocal microscope using 20× DIh20 objective at 3× zoom. A single Z‐plane across the middle of a nuclei was used for CCP analysis. The nuclei tiff files were run through a MATLAB script^61^ to yield CCP values.

### 5.6 ATAC sequencing library preparation

The omni ATAC libraries were prepared using a modified omni‐ATAC protocol.^29^ Biological replicates (stiff and soft pairs) were processed on different dates. Approximately 50,000 cells were used for each transposition reaction. Reactions took place with cells attached to hydrogels. A 300 μl total volume per 12‐well plate well reaction was prepared as follows: 150 μl Tagment DNA Buffer (Illumina Ref 15027866), 7.5 μl Tagment DNA Enzyme 1 (Illumina Ref 15027865), 3 μl digitonin (Promega Ref G944A, diluted 1:1 with water), 30 μl Tween‐20, 10.5 μl water, 89 μl PBS, incubating for 50 min at 37°C. The pre‐amplified transposed fragments were extracted from the hydrogel‐attached cells with a phenol/chloroform/isoamyl alcohol (25:24:1) DNA precipitation. The postamplified ATAC‐libraries were cleaned‐up with DNA Clean and Concentrator‐5 Kit (Zymo Research Ref D4014). The qPCR amplification was done using NEBNext Ultra II Q5 Master Mix (NEB Ref M0544S), SYBR Gold (Life Tech Ref S11494), and Nextera DNA CD Indices (Illumina Ref 20015882). The libraries were size selected to remove DNA fragments greater than 1000 bp with a Sage Science Blue Pippin. The ATAC‐seq libraries were quantified with Qubit HS DNA assay. The libraries fragment size distributions determined with Agilent HS D5000 ScreenTape.

### 5.7 ATAC‐seq processing and differential accessibility analysis

Libraries were pooled and sequenced as paired‐end 2 × 37 reads with a NextSeq sequencer at the University of Colorado Boulder BioFrontiers Next Generation Sequencing Core. ATAC‐seq datasets were trimmed with bbduk (version 38.05) to remove adapters. Quality was checked using fastQC (version 0.11.5). Fastq datasets were aligned to the SusScrofa 11.1 genome using hisat2 (version 2.1.0). Samtools (version 1.10) was used to convert SAM files to sorted BAM files and to remove PCR duplicates. ATAC‐seq peaks were called using MACS2 (version 2.1.1.20160309). ATAC‐seq datasets were analyzed for differential accessibility between stiff and soft hydrogels using Bioconductor package DiffBind (2.14.0) and an adjusted *p*‐value of <0.05 was used as a cutoff and saved as bed files. Peaks were separated by increased or decreased accessibly on stiff hydrogels for further analysis (greater or less than 0 fold‐change). Heatmaps were generated using pheatmap (1.0.12). Differentially accessible regions were annotated and visualized using ChIPseeker (version 1.22.1)^62^ and clusterProfiler (version 3.14.3).^63^ Peaks were associated with genes if their annotation was not distally intergenic using the UCSC Sus Scrofa 11 refGene (TxDb.Sscrofa.UCSC.susScr11.refGene) using ChIPseeker.

### 5.8 ATAC‐seq binding motif enrichment

Homer (version 4.11)^64^ was used for finding enriched motifs in differentially accessible genomic regions separated by increased accessibility on soft or stiff hydrogels. The size parameter for motif finding used was 200 with masking. The merged samples MACS2 narrowpeak files were used as a custom background to correct for cell type specific sequence enrichment. Homer de novo Motif Results were reported for the custom background (Figure 2) and known motifs were reported for all peaks found in stiff and soft samples, and differentially accessible regions (Table S1, S2, S4, S5).

Additionally, to look for differential transcription factor activity from the ATAC datasets from the cells cultured on stiff or soft hydrogels, we used the transcription factor expression analysis (TFEA)^33^ (Table S3). The transcription factor activity was assessed with the JASPAR2020_CORE_vertebrates_non‐redundant motif database.^65^ TFEA estimates enrichment scores on the co‐occurrence of transcription factor motifs with ATAC‐Seq peaks and then estimates a change in this score across stiff versus soft datasets per transcription factor as a surrogate for differential transcription factor activity.

### 5.9 RNA isolation, sequencing, and differential expression analysis

Cells were cultured on stiff or soft hydrogels (25 mm^2^) for 3 days. The Qiagen RNAeasy kit (Cat# 74106) was used for RNA isolation. RNA was evaluated for quality using Agilent 2100 Bioanalyzer. Libraries were generated using KAPA HyperPrep mRNA kit (Kapa Biosystems). Libraries were sequenced on NextSeq 500 (Illumina) as single‐ended reads (1x75). RNA‐seq datasets were trimmed using trimmomatic (version 0.36). FastQC (version 0.11.5) was used for quality control. Hisat2 (version 2.1.0) was used to align to the SusScrofa 11.1 genome. Samtools (version 1.3.1) was used to sort and convert SAM files to BAM files. R subread function featureCounts (version 1.30.9) was used to quantify read counts for each sample. edgeR (version 3.30.3) was used for differential expression analysis with the glmQLF function with an adjusted *p* value cutoff of <0.05. pheatmap (version 1.0.12). ClusterProfiler (version 3.16.1)^63^ was used for functional analysis and visualization with the enrichGO function. The histone modification gene ontology term (GO:0016570) gene list was used to identify logCPM values across all samples. Chromatin modifiers were manually annotated with general function (acetyltransferase, etc.) and target modifications using genecards.org.

### 5.10 Correlation of ATAC‐Seq and RNA‐seq

ChIPseeker (version 1.22.1)^62^ and clusterProfiler (version 3.14.3)^63^ were used for functional enrichment analysis. Promoters were defined if within 5000 bp of the transcription start site of a gene. The overlap between the upregulated (RNA‐seq) and increased accessible (ATAC‐seq) genes were determined and plotted using the euler package. A hypergeometric distribution test (phyper) was used to determine if the overlap was significant. enrichGO function of the clusterProfiler package was used for GO term enrichment. The fibrosis associated values using Harmonizome Fibrosis CTD Gene‐Disease Associations Gene Set^34^ were found for each gene found in the overlap between ATAC and RNA‐Seq datasets on stiff hydrogels. Genes were associated with fibrosis if their fibrosis association value was >2.

### 5.11 Human aortic valve tissue analysis

Patient information can be found in Table S10. Several samples were obtained from Origene as frozen tissue sections from the aortic valve. The remaining aortic valve leaflet samples were obtained from human patients who were decreased or undergoing heart transplantations at the University of Colorado Anschutz Medical Campus, or from excised valve tissue obtained during surgical aortic valve replacement at the University of Iowa. Leaflets were washed in warmed 1X Earle's Balanced Salt Solution (EBSS, Sigma‐Aldrich). Leaflets were then embedded and sectioned as follows: (1) laid out flat onto a microscope glass slide using tweezers, (2) flash frozen in an isopentane (Sigma) bath chilled with dry ice, (3) embedded with optimal cutting temperature (OCT) compound (TissueTek) in cryo‐sectioning molds, and (4) sectioned using a Leica CM1850 cryostat at 10 μm thickness onto Superfrost Plus microscope slides (Fisher Scientific). Sections were stored in a −20°C freezer prior to staining.

### 5.12 Proliferation characterization

To assess proliferation, an Alexa Fluor Click‐iT 5‐ethynyl‐2′‐deoxyuridine (EdU) Cell Proliferation Kit (Thermo Fisher) was used. Prior to fixing cells were treated with 10 μmol/ml EdU for 24 h. Cells cultured for a total of 3 days and were fixed and permeabilized as described above and stained with 1:5000 dilution of HCS Cell Mask (Life Technologies), and 1:1000 dilution of DAPI (Life Technologies) for 1 h. The Click‐iT reaction was then performed according to manufacturer's instructions. Cells were imaged using Operetta as described above. Harmony software was used to quantify number of cells with EdU positive nuclei. At least 380 cells were quantified for each replicate.

Frozen sections were stained for CREBBP using the HRP/DAB (ABC) Detection Kit (ab64261). Briefly, sections were fixed with 4% paraformaldehyde (Electron Microscopy Sciences 15710) for 15 min and permeabilized in 0.01% Triton X100 for 1 h at room temperature. Sections were blocked in 5% BSA (Sigma BP1600) for 1 h. Sections were incubated overnight at 4°C with anti‐CREBBP primary antibody in 5% BSA (abcam ab2832, 1:1000). Sections were then washed 4× in PBS, and biotinylated goat anti rabbit IgG(H + L) was applied and incubated for 10 min at room temperature. The sectioned were washed 4× with PBS, then streptavidin peroxidase was applied and incubated for 10 min at room temperature. DAB solution was applied to the section and incubated for 5 min. Sections were counterstained for hematoxylin (Thermo Fisher) for 2 min and rinsed in water. Sections were imaged using a Nikon Eclipse TE300 and a color camera. Images were quantified using FIJI to measure integrated image intensity. Briefly, images were converted into gray scale and the entire tissue was selected using the lasso tool. The integrated image intensity was quantified using the measure function and used for statistical analysis.

### 5.13 Statistics

Data in bar graphs are presented as mean ± standard error of mean with a minimum of three biological and technical replicates for all studies unless otherwise stated. Violin plots were used to represent single cell data. Significance was claimed at *p < 0.05, **p < 0.01, ***p < 0.005, ****p < 0.001 using a *t*‐test for comparison for normal distribution and Wilcoxon signed rank test for non‐normal distributions of two samples and one‐way analysis of variance (ANOVA) for comparison of three and more samples using GraphPad Prism.

## AUTHOR CONTRIBUTIONS

**Dilara Batan:** Conceptualization (supporting); data curation (equal); formal analysis (equal); investigation (equal); methodology (equal); validation (equal); visualization (equal); writing – original draft (equal); writing – review and editing (lead). **Carrie Bishop:** Formal analysis (supporting); visualization (supporting); writing – review and editing (supporting). **Daniel Ramirez:** Data curation (supporting); formal analysis (supporting); methodology (supporting); writing – review and editing (supporting). **Brian A. Aguado:** Resources (supporting); validation (supporting); writing – review and editing (supporting). **Megan E. Schroeder:** Data curation (supporting); writing – review and editing (supporting). **Claudia Crocini:** Writing – original draft (supporting); writing – review and editing (supporting). **Jessica Schwisow:** Resources (supporting). **Karen Moulton:** Resources (supporting). **Laura Macdougall:** Resources (supporting); writing – review and editing (supporting). **Robert M. Weiss:** Resources (supporting); writing – review and editing (supporting). **Mary A. Allen:** Methodology (supporting); supervision (supporting). **Robin Dowell:** Methodology (supporting); supervision (supporting); writing – review and editing (supporting). **Cierra J. Walker:** Conceptualization (lead); investigation (equal); methodology (equal); validation (equal); visualization (equal); writing – review and editing (supporting). **Kristi S. Anseth:** Conceptualization (supporting); resources (supporting); supervision (supporting); writing – review and editing (supporting). **Leslie A. Leinwand:** Conceptualization (supporting); resources (supporting); supervision (supporting); writing – review and editing (supporting).

## CONFLICT OF INTEREST

The authors declare no conflicts of interests.

## Supporting information


**Supplementary Figure 1 (A)** Young's elastic modulus of soft and stiff hydrogels. *n* = 6 hydrogels. Paired two‐way student's *t*‐test applied. (B) YAP nuclear to cytoplasm intensity for VICs cultured on soft or stiff hydrogels. *n* ≥ 7 hydrogels. Unpaired two‐way student's *t*‐test applied. (C) H3ac nuclear intensity in VICs cultured on soft, stiff + verteporfin (YAP inhibitor) (1 μM), or stiff hydrogels. *n* > 223 cells across four hydrogels. One‐way ANOVA with Bonferroni posthoc test applied. Data shown as mean ± SEM.
**Supplementary Figure 2:** (A) Significant GO terms identified for overlapped genes between upregulated and increased accessible genes for VICs on soft hydrogels. (B) RNA‐Seq fold change, ATAC‐Seq fold change, and fibrosis association value of genes upregulated and with increased accessibility for VICs on soft hydrogels. (C) Pie charts illustrating the fraction of overlapped genes from ATAC‐Seq and RNA‐Seq that are linked to fibrosis (defined as fibrosis association value > 2).
**Supplementary Figure 3:** Proliferation of VICs on soft and stiff hydrogels measured by Ki67 nuclear positive staining. *n* = 6 hydrogels. Unpaired, two‐way student's *t*‐test applied. Data shown as mean ± SEM.
**Supplementary Figure 4:** Nuclear KDM6B expression in VICs cultured on soft or stiff hydrogels. *n* > 114 cells across two hydrogels. Unpaired, two‐way student's *t*‐test applied.
**Supplementary Figure 5:** (A) VICs on TCPS treated with EX527 SIRT1 inhibitor and (B) SGC‐CBP30 (CBP30) CREBBP inhibitor at concentrations known to inhibit proteins and measured for cell number, αSMA intensity, and nuclear H3ac intensity. *n* ≥ 4 wells. (C) H3ac and CCP quantification for VICs cultured on soft, soft + CBP‐30 (CREBBP inhibitor), or stiff hydrogels. *n* = cells. One‐way ANOVA with Bonferroni post hoc test applied. Data shown as mean ± SEM.
**Supplementary Figure 6:** TTK21 CREBBP inhibitor at concentrations known to inhibit proteins and measured for (A) cell number and (B) αSMA intensity *n* ≥ 4 well. One‐way ANOVA with Bonferroni post hoc test applied. Data shown as mean ± SEM.
**Supplementary Figure 7:** (A) Representative images of EdU staining on soft hydrogels treated with DMSO or CBP‐30 and stiff hydrogels treated with DMSO. Scale bar = 100 μm. (B) Quantification of percent cells with positive EdU staining for A. One‐way ANOVA with Bonferroni post hoc test applied (*n* ≥ 4 hydrogels means ± SEM shown, *p < 0.05).

**Supplementary Table 1** Homer known motifs: Regions with increased accessibility
**Supplementary Table 2**. Homer known motifs: Regions with increased accessibility
**Supplementary Table 3**. TFEA transcription factors
**Supplementary Table 4**. Homer known motifs
**Supplementary Table 5**. Homer known motifs
**Supplementary Table 6**. Annotated differentially accessible regions
**Supplementary Table 7**. Annotated differentially accessible regions
**Supplementary Table 8**. Differentially expressed genes
**Supplementary Table 9**. Overlap of ATAC & RNA (annotated NOT with distal intergenic)
**Supplementary Table 10**. Overlap of ATAC & RNA (annotated NOT with distal intergenic)
**Supplementary Table 11**. Patient Samples

## Data Availability

The authors declare that the main data supporting the results in this study are available within the paper and its Supplementary Information (SI). ATAC‐Seq and RNA‐Seq data generated in this study are available through the Gene Expression Omnibus under accession code (not available yet but will be before publication). Additional datasets are available from the corresponding author upon reasonable request.
