# Supplementary material for: Extracellular matrix stiffness controls cardiac valve myofibroblast activation through epigenetic remodeling
Source: Bioeng Transl Med. 2022 Aug 22;7(3):e10394. doi: 10.1002/btm2.10394 (PMC9472021; doi:10.1002/btm2.10394)
Supplement: Supplementary file 1 — Supplementary Figure 1 (A) Young's elastic modulus of soft and stiff hydrogels. n = 6 hydrogels. Paired two‐way student's t‐test applied. (B) YAP nuclear to cytoplasm intensity for VICs cultured on soft or stiff hydrogels. n ≥ 7 hydrogels. Unpaired two‐way student's t‐test applied. (C) H3ac nuclear intensity in VICs cultured on soft, stiff + verteporfin (YAP inhibitor) (1 μM), or stiff hydrogels. n > 223 cells across four hydrogels. One‐way ANOVA with Bonferroni posthoc test applied. Data shown as mean ± SEM. Supplementary Figure 2: (A) Significant GO terms identified for overlapped genes between upregulated and increased accessible genes for VICs on soft hydrogels. (B) RNA‐Seq fold change, ATAC‐Seq fold change, and fibrosis association value of genes upregulated and with increased accessibility for VICs on soft hydrogels. (C) Pie charts illustrating the fraction of overlapped genes from ATAC‐Seq and RNA‐Seq that are linked to fibrosis (defined as fibrosis association value > 2). Supplementary Figure 3: Proliferation of VICs on soft and stiff hydrogels measured by Ki67 nuclear positive staining. n = 6 hydrogels. Unpaired, two‐way student's t‐test applied. Data shown as mean ± SEM. Supplementary Figure 4: Nuclear KDM6B expression in VICs cultured on soft or stiff hydrogels. n > 114 cells across two hydrogels. Unpaired, two‐way student's t‐test applied. Supplementary Figure 5: (A) VICs on TCPS treated with EX527 SIRT1 inhibitor and (B) SGC‐CBP30 (CBP30) CREBBP inhibitor at concentrations known to inhibit proteins and measured for cell number, αSMA intensity, and nuclear H3ac intensity. n ≥ 4 wells. (C) H3ac and CCP quantification for VICs cultured on soft, soft + CBP‐30 (CREBBP inhibitor), or stiff hydrogels. n = cells. One‐way ANOVA with Bonferroni post hoc test applied. Data shown as mean ± SEM. Supplementary Figure 6: TTK21 CREBBP inhibitor at concentrations known to inhibit proteins and measured for (A) cell number and (B) αSMA intensity n ≥ 4 well. One‐way ANOVA [file BTM2-7-e10394-s002.docx]

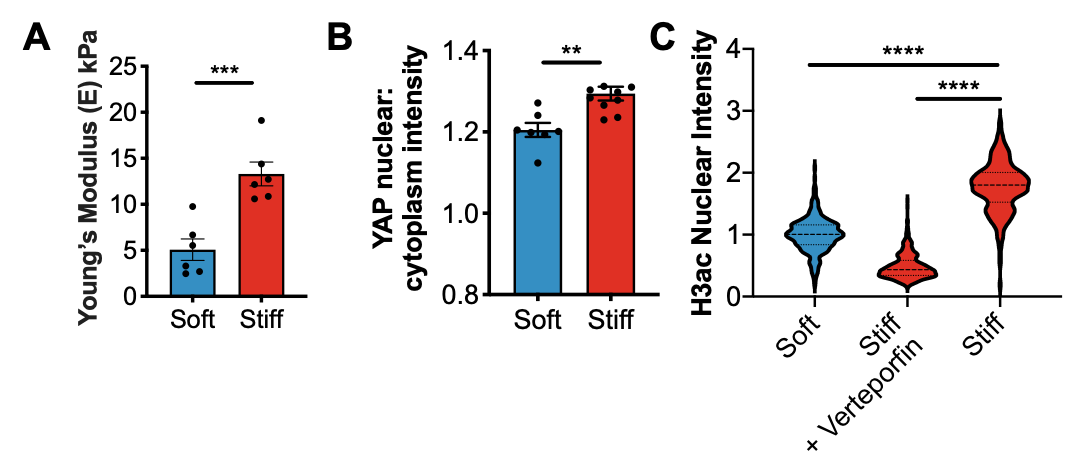


**Supplementary Figure 1: A)** Young’s elastic modulus of soft and stiff hydrogels. n=6 hydrogels. Paired two-way student’s t-test applied. B) YAP nuclear to cytoplasm intensity for VICs cultured on soft or stiff hydrogels. n>7 hydrogels. Unpaired two-way student’s t-test applied. C) H3ac nuclear intensity in VICs cultured on soft, stiff + verteporfin (YAP inhibitor) (1 uM), or stiff hydrogels. n>223 cells across 4 hydrogels. One-way ANOVA with Bonferroni posthoc test applied. Data shown as mean +/- SEM.


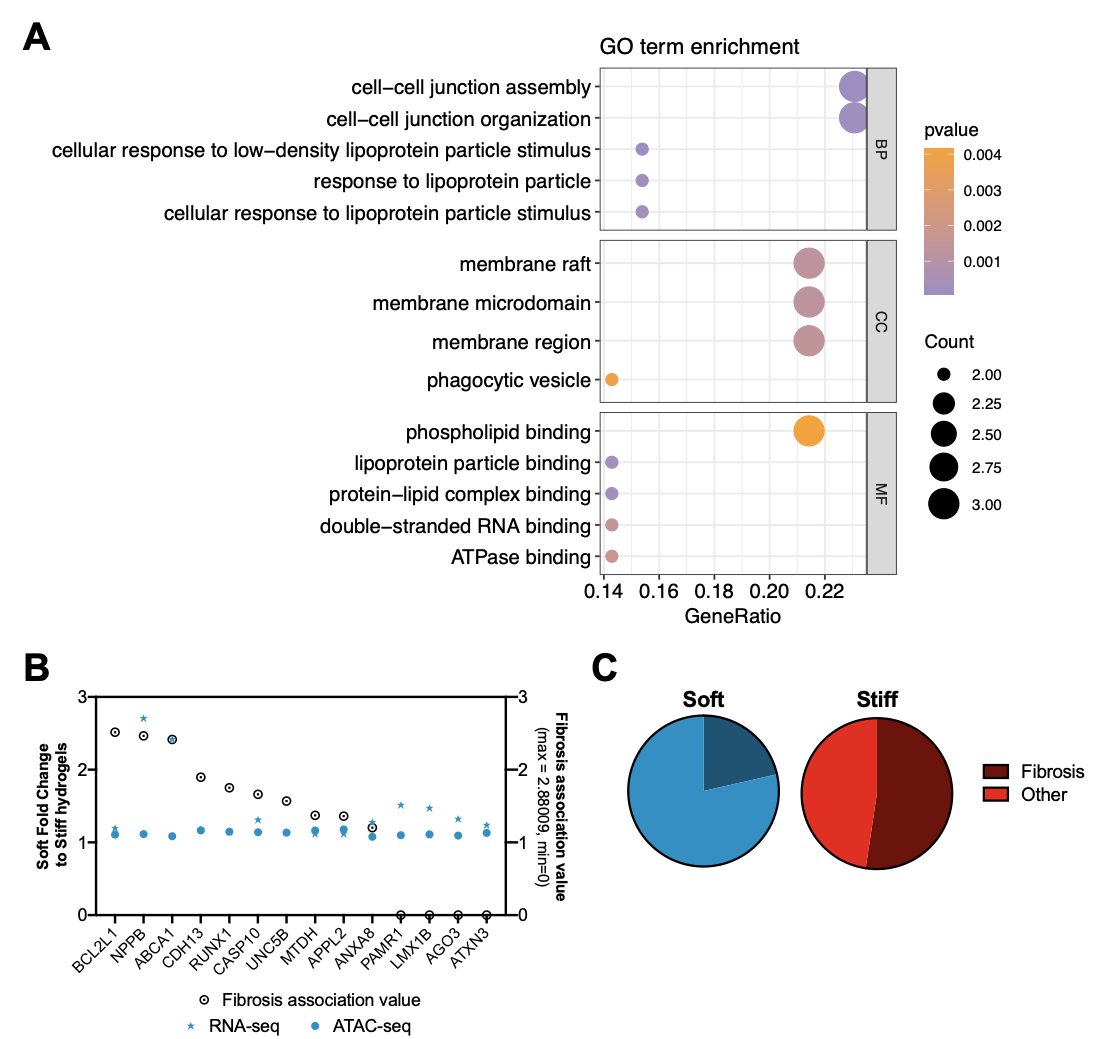


**Supplementary Figure 2:** A) Significant GO terms identified for overlapped genes between upregulated and increased accessible genes for VICs on soft hydrogels. B) RNA-Seq fold change, ATAC-Seq fold change, and fibrosis association value of genes upregulated and with increased accessibility for VICs on soft hydrogels. C) Pie charts illustrating the fraction of overlapped genes from ATAC-Seq and RNA-Seq that are linked to fibrosis (defined as fibrosis association value > 2).


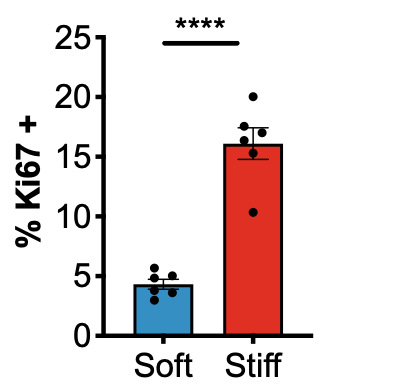


**Supplementary Figure 3:** Proliferation of VICs on soft and stiff hydrogels measured by Ki67 nuclear positive staining. n=6 hydrogels. Unpaired, two-way student’s t-test applied. Data shown as mean +/- SEM.


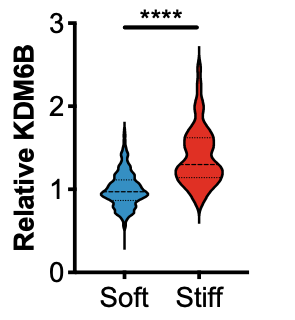


**Supplementary Figure 4:** Nuclear KDM6B expression in VICs cultured on soft or stiff hydrogels. n>114 cells across 2 hydrogels. Unpaired, two-way student’s t-test applied.


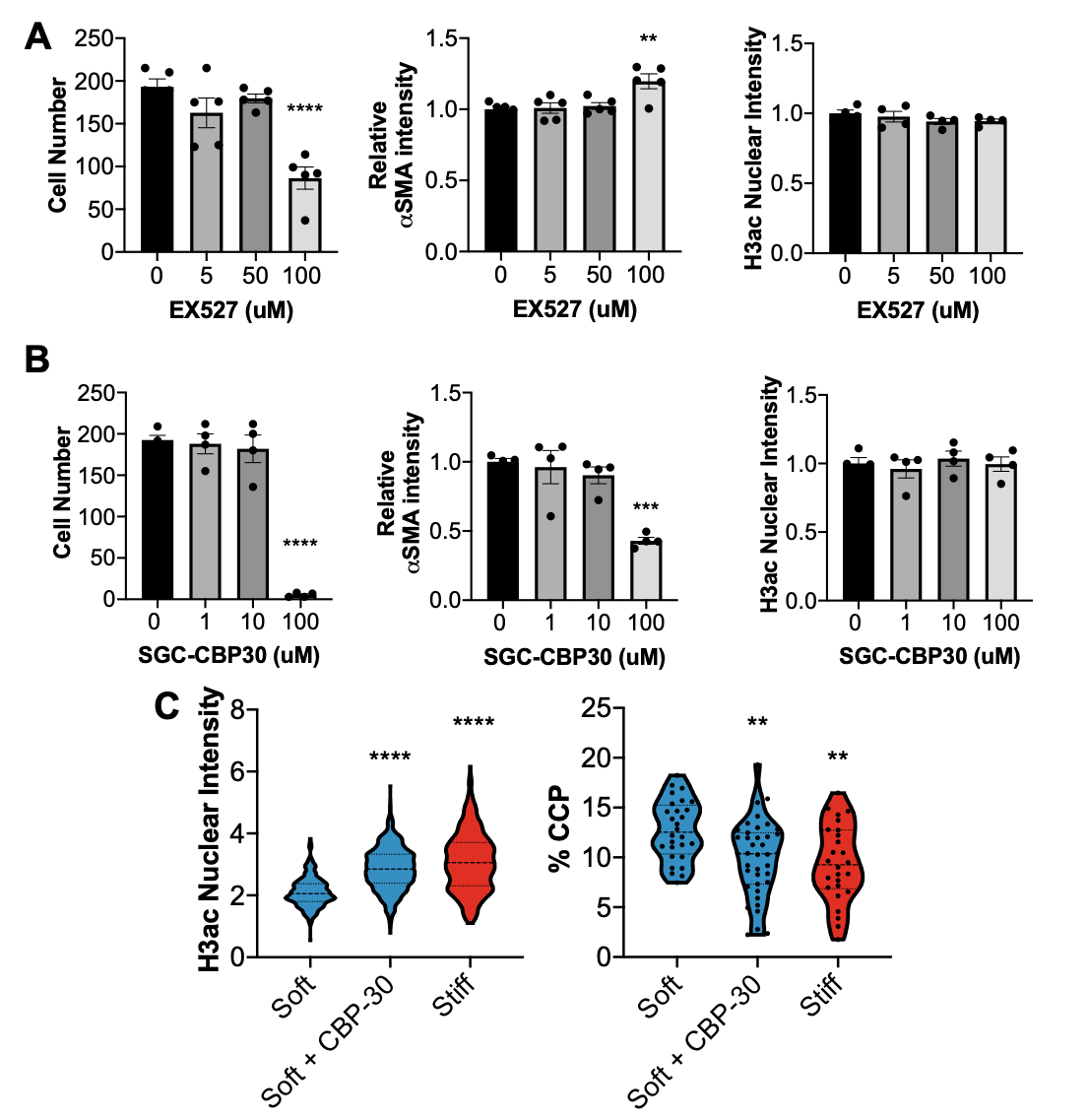


**Supplementary Figure 5:** A) VICs on TCPS treated with EX527 SIRT1 inhibitor and (B) SGC-CBP30 (CBP30) CREBBP inhibitor at concentrations known to inhibit proteins and measured for cell number, αSMA intensity, and nuclear H3ac intensity. n>4 wells. C) H3ac and CCP quantification for VICs cultured on soft, soft + CBP-30 (CREBBP inhibitor), or stiff hydrogels. n=cells. One-way ANOVA with Bonferroni posthoc test applied. Data shown as mean +/- SEM.

   
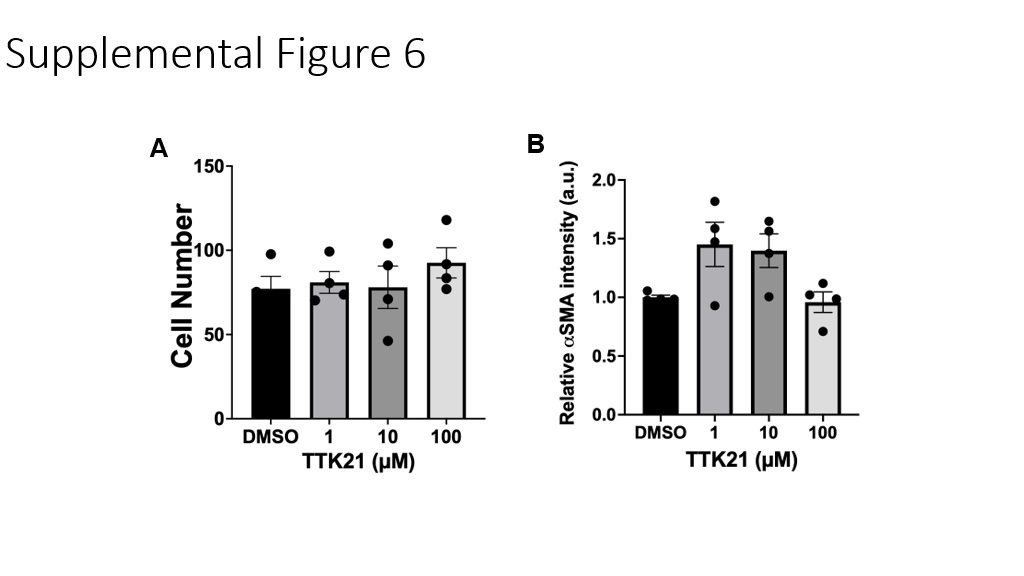


**Supplementary Figure 6:** TTK21 CREBBP inhibitor at concentrations known to inhibit proteins and measured for A) cell number and B) αSMA intensity n>4 well. One-way ANOVA with Bonferroni posthoc test applied. Data shown as mean +/- SEM.

**
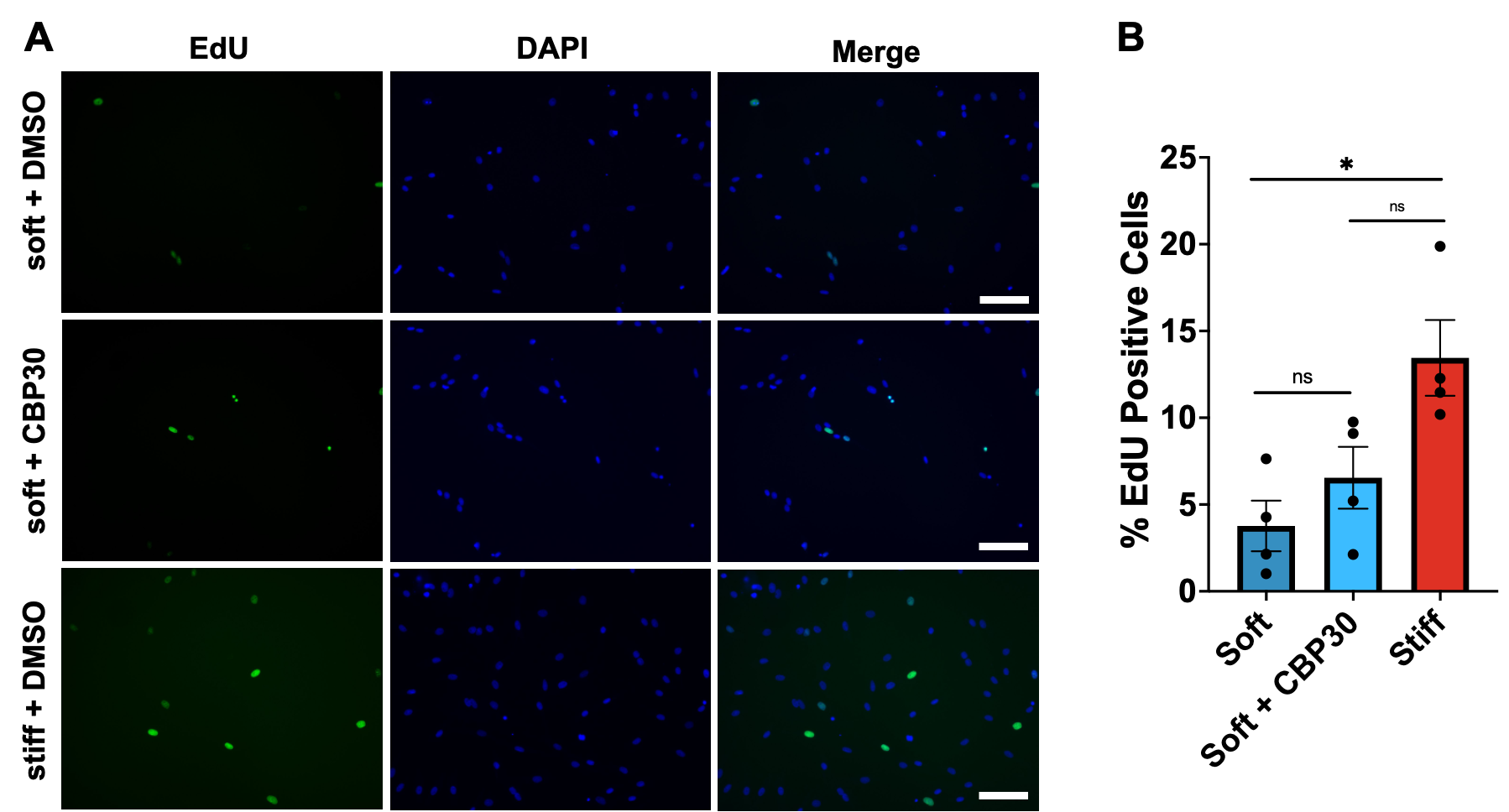
**

**Supplementary Figure 7:** A) Representative images of EdU staining on soft hydrogels treated with DMSO or CBP-30 and stiff hydrogels treated with DMSO. Scale bar = 100 µm. B) Quantification of percent cells with positive EdU staining for A. One-way ANOVA with Bonferroni posthoc test applied (n ≥ 4 hydrogels means ± SEM shown, *p < .05)
